# The biological role and immunotherapy of gangliosides and GD3 synthase in cancers

**DOI:** 10.3389/fcell.2023.1076862

**Published:** 2023-02-07

**Authors:** Shangqi Cao, Xu Hu, Shangqing Ren, Yaohui Wang, Yanxiang Shao, Kan Wu, Zhen Yang, Weixiao Yang, Gu He, Xiang Li

**Affiliations:** ^1^ Department of Urology, Institute of Urology, West China Hospital, West China Medical School, Sichuan University, Chengdu, China; ^2^ Robotic Minimally Invasive Surgery Center, Sichuan Academy of Medical Sciences and Sichuan Provincial Peoples Hospital, Chengdu, China; ^3^ Department of Urology, Chengdu Second People’s Hospital, Chengdu, China; ^4^ State Key Laboratory of Biotherapy and Department of Pharmacy, West China Hospital, Sichuan University and Collaborative Innovation Center of Biotherapy, Chengdu, Sichuan, China

**Keywords:** gangliosides, GD2, GD3, GD3 synthase, cancer biology, immunotherapy

## Abstract

Gangliosides are a large subfamily of glycosphingolipids that broadly exist in the nervous system and interact with signaling molecules in the lipid rafts. GD3 and GD2 are two types of disialogangliosides (GDs) that include two sialic acid residues. The expression of GD3 and GD2 in various cancers is mostly upregulated and is involved in tumor proliferation, invasion, metastasis, and immune responses. GD3 synthase (GD3S, ST8SiaI), a subclass of sialyltransferases, regulates the biosynthesis of GD3 and GD2. GD3S is also upregulated in most tumors and plays an important role in the development and progression of tumors. Many clinical trials targeting GD2 are ongoing and various immunotherapy studies targeting gangliosides and GD3S are gradually attracting much interest and attention. This review summarizes the function, molecular mechanisms, and ongoing clinical applications of GD3, GD2, and GD3S in abundant types of tumors, which aims to provide novel targets for future cancer therapy.

## 1 Introduction

Cancer is a major public health burden in the world, with an estimated 19.3 million new cancer cases and almost 10.0 million cancer deaths occurring in 2020 (Sung et al., 2021). Although the relevant screening, targeted therapy, and immunotherapy have been proposed and widely applied, leading to a significantly improved cancer prognosis for patients, cancer-related deaths are still increasing and cause a huge burden on public health. Therefore, it is necessary to explore novel targets for cancer therapies.

**FIGURE 1:**
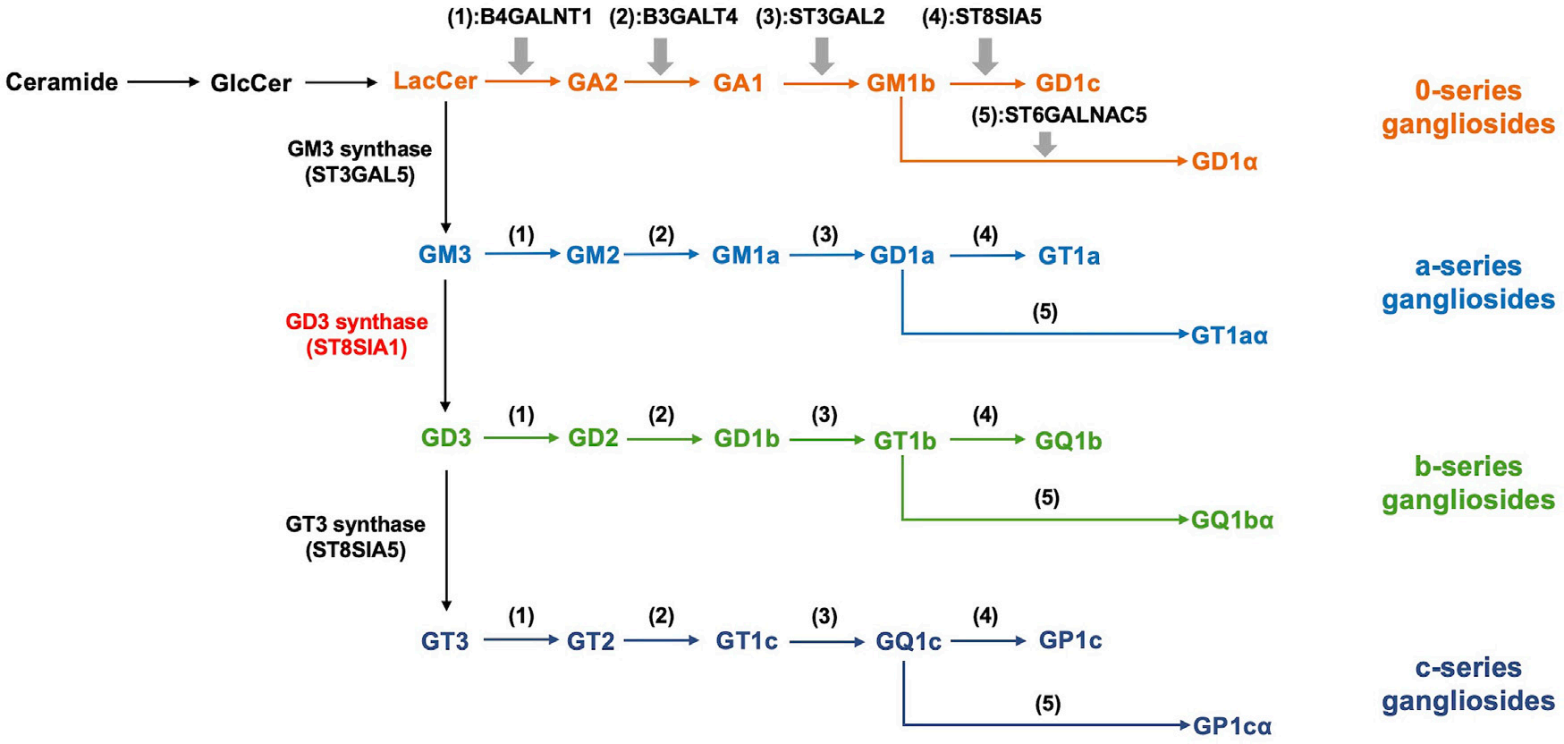
Biosynthesis and classification of gangliosides. The synthesis of gangliosides presents the stepwise addition of monosaccharides with ceramides. ST3Gal V, ST8Sia I, and ST8Sia V catalyze the synthesis of precursors of the a, b, and c-series gangliosides, respectively. 0-series gangliosides are synthesized directly from LacCer with enzymatic catalysis. B4GALNT1, B3GALT4, ST3GAL2, ST8SIA5, and ST6GALNAC5 catalyze the synthesis of various members of 0, a, b, and c-series gangliosides in turn. According to the number of sialic acid residues, gangliosides can be mainly classified into monosialogangliosides (GM), disialogangliosides (GD), trisialogangliosides (GT) and tetrasialoganglioside (GQ).

Sialic acids are a family of negatively charged monosaccharides with a nine-carbon backbone that commonly occur in deuterostomes and some microorganisms (Angata and Varki, 2002; Li and Chen, 2012; Teppa et al., 2016; Yang et al., 2021). Sialic acids are composed of more than 50 different compounds including N-acetylneuraminic acids (Neu5Ac), N-glycolyl neuraminic acids (Neu5Gc), and 2-keto-3-deoxynononic acids (KDN) (Schauer and Kamerling, 2018; Yang et al., 2021). The surface of eukaryotic cells is covered by a large number of sialylated molecules, which regulate various physiological activities of the human body including immunoregulation, cell migration, metastasis, adhesion, invasion, and cell-cell interactions (Takashima et al., 2013; Teppa et al., 2016). Gangliosides are a large subclass of glycosphingolipids containing one or more sialic acid residues on their carbohydrate moiety, comprising a hydrophobic ceramide and a hydrophilic oligosaccharide chain with sialic acids (Groux-Degroote et al., 2017; Liu et al., 2018). Ernst Klenk discovered gangliosides in the *postmortem* brain tissue of infant patients when he had a study on amaurotic familial idiocy. He named the term “gangliosides” based on the abundance in ganglia (Akkhawattanangkul et al., 2017; Sandhoff and Sandhoff, 2018). Gangliosides are enriched in lipid rafts which are heterogeneous and highly dynamic microdomains of cell membrane rich in cholesterol and sphingolipids (Pike, 2006). Even if gangliosides are the minor constituents of eukaryotic cell membranes, they are relatively plentiful in certain cell types and tissues and are especially expressed at a high level in the nervous system (Groux-Degroote et al., 2018; Sonnino et al., 2018; Sipione et al., 2020). It has been demonstrated that gangliosides are involved in normal neural development and function *via* regulating signal transduction (Schengrund, 2015). In addition, gangliosides were considered to be essential in the protection of neurodegeneration, the regulation of the complement system, and the maintenance of nerve tissues (Ohmi et al., 2009; Ohmi et al., 2011). Based on the Svennerholm nomenclature, gangliosides are categorized as four series including 0-, a-, b-, and c (from 0 to 3 sialic acids linked to the inner galactose residue). According to the number of sialic acid residues, gangliosides can be mainly classified into monosialogangliosides (GM), disialogangliosides (GD), trisialogangliosides (GT) and tetrasialoganglioside (GQ) (Svennerholm, 1980; Liu et al., 2018) (Figure 1).

GDs are members of the b-series gangliosides and possess two sialic acid residues per molecule. Gangliosides GD3 and GD2 are mainly expressed across the central nervous system (CNS), peripheral nerve tissue, and lymphocytes (Cavdarli et al., 2019). Additionally, GD3 and GD2 are considered as tumor-associated antigens, and their expression levels are mostly upregulated in cancers. The expression of GD2 is primarily concentrated on melanomas, neuroblastomas, and various other cancers including breast cancers, small cell lung cancers (SCLC), bladder cancers, and osteosarcoma (Thurin et al., 1986; Yoshida et al., 2001; Roth et al., 2014; Orsi et al., 2017; Vantaku et al., 2017; Aygün et al., 2019). GD3 is usually expressed to a variable degree in gliomas, melanomas, and breast cancers(Yeh et al., 2016; Liang et al., 2017; Ohmi et al., 2018). It has been shown that GD3 and GD2 have an impact on tumor progression and various malignant properties of tumor cells, which attracts various studies of immunotherapies targeting GD3 and GD2 (Thurin et al., 1986; Yoshida et al., 2001; Yeh et al., 2016; Liang et al., 2017; Orsi et al., 2017; Vantaku et al., 2017; Ohmi et al., 2018; Aygün et al., 2019).

**FIGURE 2:**
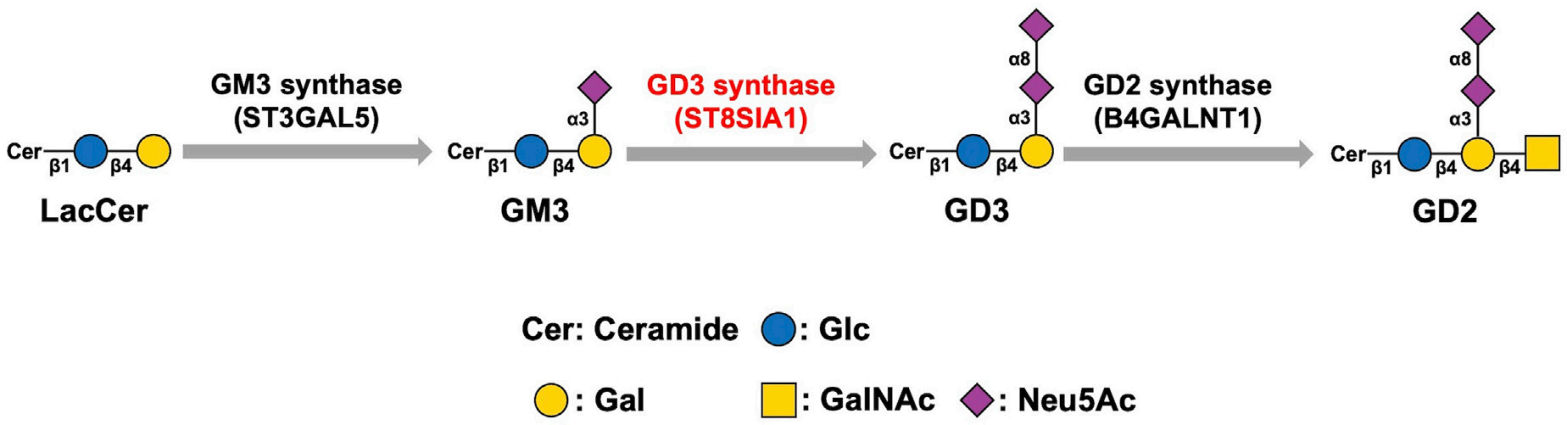
Biosynthesis pathway of GD3 and GD2. GD3 synthase catalyzes the synthesis of GD3 from GM3. GD3 is converted to GD2 *via* the catalysis of GD2 synthase. Therefore, GD3S is the key enzyme that can control both gangliosides GD3 and GD2 biosynthesis.

The mammalian sialyltransferase (ST) family includes 20 STs. Sialylation of glycoconjugates is catalyzed by the ST family. Based on the distinct types of glycosidic linkages formed, the ST family is classified into four members, namely ST3Gal (α2,3-ST), ST6Gal (α2,6-ST), ST6GalNAc, and ST8Sia (α2,8-ST). The family members catalyze the transfer of sialic acid from CMP-Neu5Ac to the terminal position of glycoproteins and glycolipids to synthesize sialylated glycoconjugates (Harduin-Lepers et al., 2005; Bhide and Colley, 2017; Huang et al., 2017). The ST8Sia family includes ST8Sia I, II, III, IV, V, and VI, the six members of which are categorized into three varieties: Mono-STs (ST8Sia I, V, and VI), oligo-ST (ST8Sia III), and poly-STs (ST8Sia II and IV). The ST8Sia family can catalyze the sialylation of glycoconjugates *via* the addition of sialic acids from CMP-NeuAc to the underlying glycan chains for the formation of sialylated molecules (Huang et al., 2017). ST8Sia I can directly synthesize GD3 by transferring sialic acid residues from CMP-NeuAc to GM3. Therefore, ST8Sia I is also referred to as GD3 synthase (GD3S). GD2 is derived from GD3 through the catalysis of GD2 synthase (Figure 2). Thus it can be seen that GD3S is the key enzyme that can control both ganglioside GD3 and GD2 biosynthesis (Liu et al., 2018). GD3S was highly expressed in the early stage of development whereas after birth the expression of GD3S was low and almost undetectable in brain of adults except in choroid plexus (Yamamoto et al., 1996; Yamada et al., 2005). Moreover, many studies have proven that GD3S exerts great significance on the proliferation, invasion, and metastasis of malignant tumors and is considered a novel tumor-associated target for immunotherapy (Cazet et al., 2009; Lluis et al., 2009; Cazet et al., 2010; Kwon et al., 2010).

In this review, we summarize the related knowledge and results about the expression, biological functions, and immunotherapy studies of disialogangliosides GD3 and GD2 as well as GD3S in cancers, providing the possibility of gangliosides and GD3 synthase as therapeutic targets in cancer treatment.

## 2 Gangliosides

### 2.1 The biological functions of gangliosides in cancers

Gangliosides are often highly expressed in a variety of tumor cells, and the biological functions of gangliosides in cancers have been explored widely by numerous investigations. Gangliosides play a role as signal regulators in cancers *via* interaction with different types of receptor tyrosine kinases in lipid rafts of tumor cells to activate or suppress various signal pathways, resulting in regulating tumor progression and malignant characteristics of tumor cells. The change in signal transduction affected by gangliosides is closely related to the development of tumor cell properties such as cell proliferation, invasion, and apoptosis. (Sasaki et al., 2021). Additionally, gangliosides expressed in tumor cells may shed from their membrane into tumor microenvironment, including tumor cells in YAC-1 lymphoma (Ladisch et al., 1983), melanoma (Bernhard et al., 1989), medulloblastoma (Chang et al., 1997), neuroblastoma (Valentino et al., 1990), ovarian cancer (Dyatlovitskaya et al., 1997), gliomas (Nakamura et al., 1991), and retinoblastoma (Portoukalian et al., 1993). Shedding tumor gangliosides exert potent significance on immunosuppression in tumor microenvironment. Shen et al. indicated that gangliosides prevented the maturation of dendritic cells (DCs) induced by lipopolysaccharide, which may have the suppressive effects on the anti-tumor immune response (Shen and Ladisch, 2002). Caldwell et al. demonstrated that gangliosides had a suppressive impact on the biological function of antigen-presenting cells including monocytes and DCs, resulting in inhibiting the proliferation of T cells (Caldwell et al., 2003). DCs exposed to gangliosides was shown to promote the higher regulatory T-cell activity, leading to inhibiting T-cell effector roles (Jales et al., 2011). Gangliosides shed from tumors suppressed the cytotoxicity of CD8^+^ T cells by inhibition of lytic granule trafficking and interference with granule exocytosis (Lee H. C. et al., 2012). Moreover, gangliosides also play various important roles in tumor angiogenesis (Liu et al., 2006; Liu et al., 2014), cell adhesion (Ohkawa et al., 2010), and metastasis (Ramos et al., 2020). Mukherjee et al. demonstrated that ganglioside GM3 played an antiangiogenic role, reversing the proangiogenic effects of vascular endothelial growth factor and GD1a (Mukherjee P. et al., 2008). Zeng et al. discovered that tumor angiogenesis was inhibited with suppression of GD3 expression (Zeng et al., 2000). Moreover, Tumor angiogenesis was activated or inhibited with the decrease or increase of the GM3:GD3 ratio respectively (Ziche et al., 1992).

From the various biological roles of gangliosides in tumors, it can be seen that regulation of ganglioside expression is very important for controlling tumor progression and immune response. In addition to the regulatory effects of STs on the expression of gangliosides, the horizontal transfer of extracellular gangliosides also plays a crucial role in tumor microenvironment. Gangliosides have been demonstrated to be enriched and localized in membrane vesicles shed from tumor cells (Dolo et al., 2000). Otake et al. showed that extracellular vesicles enriched in GD3 shed from GD3S transfected melanoma cells transferred gangliosides from donor cells to recipient cells and promoted the migration of GD3-negative cells (Otake et al., 2019). GD3 expressed on the surface of exosome in ovarian tumor microenvironment inhibited the function of T cells (Shenoy et al., 2018). Therefore, tumor cells highly expressing gangliosides may shed extracellular vesicles enriched in gangliosides onto recipient cells to regulate the events of tumor progression and immunosuppression and promote the horizontal transfer of information even if recipient cells do not express the corresponding STs.

Disialogangliosides GD3 and GD2 as crucial b-series gangliosides have received much attention for their biological functions in tumor progression and immune system regulation. In the following content of this section, the expression and roles of GD3 and GD2 in tumors will be discussed in detail.

### 2.2 The expression and function of GD3 in cancers

#### 2.2.1 Expression of GD3 in cancers

##### 2.2.1.1 Neurological tumors

Disialoganglioside GD3 is slightly expressed in normal brains but its expression is obviously high in some pathological conditions such as many cancers and neurodegenerative disorders (Malisan and Testi, 2002). In neuroblastomas, Nishimaki et al. revealed that there were more GD3-positive and GD2-negative cells in low-risk cases than in high-risk cases (Nishimaki et al., 2021). Nakamura et al. observed that serum GD3 expression was higher in gliomas and three-grade astrocytoma specimens than in healthy and two-grade astrocytoma specimens, which indicated that the shedding of GD3 was closely related to the malignant degree of gliomas (Nakamura et al., 1991). GD3 was negative for low-grade gliomas, whereas GD3 was enriched in the perivascular area of high-grade gliomas (Koochekpour and Pilkington, 1996). Kawai et al. tested the expression of GD3 in 25 cases of astrocytomas, anaplastic astrocytomas, cerebellar astrocytomas, and glioblastomas multiforme (GM) and observed that GD3 was not expressed in normal astrocytes, whereas GD3 was expressed in neoplastic astrocytes of the above tumors (Kawai et al., 1999).

##### 2.2.1.2 Lymphoid and hematologic neoplasms

Siddiqui et al. tested the content of GD3 in acute and chronic leukemia and found that the expression of GD3 was positive in acute leukemia, whereas GD3 was not detected in chronic leukemia (Siddiqui et al., 1984). Merritt et al. tested the expression of GD3 in lymphoblasts from T-cell lymphoblastic malignancies and non-T, non-B acute lymphoblastic leukemia (ALL) pediatric cases and demonstrated that the content of GD3 was upregulated in lymphoblasts from T-cell lymphoblastic malignancies compared with non-T-cell ALL and normal tissue (Merritt et al., 1987).

##### 2.2.1.3 Other tumors

One of the most prominent gangliosides in melanomas is GD3 which regulates cell adhesion, growth, and invasion (Carubia et al., 1984; Furukawa et al., 2012; Tarhini et al., 2017). It was reported that GD3 was abundantly expressed in breast cancers, especially invasive ductal breast carcinoma (Marquina et al., 1996; Liang et al., 2017). Ye et al. found that the expression of GD3 was upregulated in human hepatomas and diethylnitrosamine-induced rat hepatomas compared to normal tissue (Ye et al., 1990). GD3 and GD2 were highly prevalent in various human sarcoma tissues. In high-grade or metastatic sarcomas, the expression of GD3 and GD2 was weakly detected (Chang et al., 1992).

#### 2.2.2 Functions and regulatory mechanisms of GD3 in cancers

**FIGURE 3:**
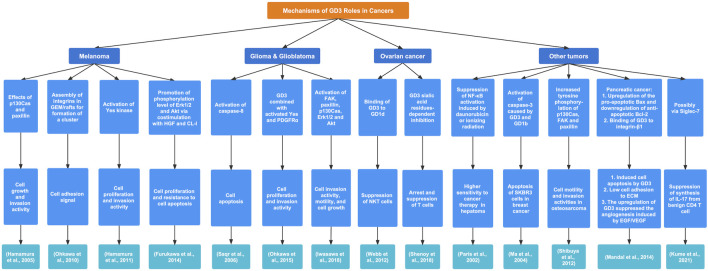
The regulatory mechanisms and biological functions of GD3 in various cancers. GD3 exerts a crucial influence on tumor progression by different signaling pathways.

Many studies have revealed that GD3 may serve as a specific antigen or target for various cancers and regulate signaling pathways and the immune environment to affect the development of tumors (Figure 3).

##### 2.2.2.1 Neurological tumors

Saqr et al. demonstrated that in a U-1242 MG glioblastoma cell line, GD3 induced cell apoptosis *via* activation of caspase-8 (Saqr et al., 2006). Ohkawa et al. discovered that in an engineered mouse model of gliomas GD3+ astrocytes had enhanced cell growth and invasion capabilities with increased phosphorylation of Akt and Yes kinase. Furthermore, GD3 combined with activated Yes and platelet-derived growth factor receptor α (PDGFRα) formed a special complex molecule, enhancing cell proliferation and invasion activity (Ohkawa et al., 2015). Increased phosphorylation levels of FAK, paxillin, p130Cas, extracellular signal-regulated kinase 1/2 (Erk1/2), and Akt were observed in the U-251MG-GD3S(+) cell lines (highly expressing GD3 and GD2), which may be related to the malignant properties of human gliomas, including significantly promoted cell invasion activity and motility as well as cell growth (Iwasawa et al., 2018).

##### 2.2.2.2 Melanoma

In melanomas, the growth and invasion activities of tumor cells were enhanced *via* GD3 with the effects of two specific molecules (p130Cas and paxillin). Therefore, the malignant phenotypes of tumor cells were suppressed by using siRNA for knockdown of p130Cas and paxillin, indicating that paxillin participated in the invasion of cells and that p130Cas participated in the growth and invasion of cells (Hamamura et al., 2005). Ohkawa et al. demonstrated that GD3+ cells in melanomas strongly adhered to ECM. In particular, when GD3+ cells were attached to collagen type I, the tyrosine phosphorylation of FAK and paxillin increased. In addition, integrins assembled in the GEM/rafts and formed a cluster under GD3 expression, which was correlated with cell adhesion. Moreover, during GD3+ cell attachment to the ECM, the phosphorylation of FAK and a signaling pathway of integrin-linked kinase/Akt were strongly activated (Ohkawa et al., 2010). In GD3+ melanoma cells, Yes, a member of the Src family kinases, was abundantly distributed in GEM/rafts and was activated through the high expression of GD3, resulting in enhancement of the malignant properties of GD3+ melanoma cells (Hamamura et al., 2011). Furukawa et al. found that the expression of GD3 in melanomas increased the phosphorylation levels of Erk1/2 and Akt for cell proliferation and resistance to cell apoptosis *via* costimulation with hepatocyte growth factor (HGF) and adhesion to collagen type I (CL-I) (Furukawa et al., 2014).

##### 2.2.2.3 Ovarian cancer

Webb et al. proved that a high concentration of GD3 in ovarian cancer-associated ascites could compete with endogenous stimulatory ligands to bind to CD1d for suppression of natural killer T (NKT) cells in ovarian cancer, which was correlated with poor prognosis (Webb et al., 2012). Shenoy et al. discovered that GD3 was detected on the surface of exosomes from ovarian tumor ascites fluids, resulting in the functional arrest of T cells *via* their TCR. In addition, It was demonstrated that GD3 was involved in the arrest and suppression of T cells even if GD3 was not localized on the inhibitory exosome, which was because the immunosuppressive ability of GD3 was closely associated with sialic acid residues (Shenoy et al., 2018). Tiper et al. found that vascular endothelial growth factor (VEGF) and GD3 could inhibit the GD1d-mediated activation of NKT cells in ovarian cancer. Additionally, the inhibition of VEGF led to the decreased expression of GD3 and reinstated the functions of NKT cells (Tiper et al., 2016).

##### 2.2.2.4 Other tumors

It was reported that the activation of NF-κB was observed in many tumor cells with chemotherapeutic treatments and ionizing radiation to inhibit cell death caused by these treatments. Paris et al. demonstrated that GD3 could suppress NF-κB activation induced by daunorubicin or ionizing radiation in human hepatoma cells, resulting in higher sensitivity to the above cancer therapies (Paris et al., 2002). In breast cancer, Ma et al. found that gangliosides GD3 and GD1b activated Caspase-3 for the apoptosis of SKBR3 cells. Moreover, the activation of caspase-3 presented a dose-dependent increase with GD3 or GD1b (Ma et al., 2004). The gangliosides GD3 and GD2 were highly expressed in osteosarcoma and promoted the malignant properties of tumor cells. In addition, GD3 and GD2 may lead to increased tyrosine phosphorylation of p130Cas, FAK, and paxillin, involved in the enhanced motility and invasion activities of osteosarcoma cells (Shibuya et al., 2012). Mandal et al. discovered that ganglioside GD3 was downregulated in pancreatic cancers. In GD3-synthase-transfected cells, GD3 induced stronger apoptosis and made cells stuck in the S-phase of the cell cycle. In addition, GD3 could bind to integrin-β1, resulting in low adhesion of cells to ECM proteins. Furthermore, the upregulation of GD3 in transfected cells suppressed the angiogenesis induced by EGF/VEGF. Therefore, the development and high case fatality rate of pancreatic adenocarcinoma may be associated with low GD3 expression (Mandal et al., 2014). In cutaneous T-cell lymphoma, the synthesis of IL-17A from benign CD4 T cells was inhibited by GD3 in part, probably through the Siglec-7 pathway (Kume et al., 2021).

#### 2.2.3 Roles of GD3 in immunomodulation

Ganglioside GD3 has an impact on immune regulation, including influencing the proliferation, activation, apoptosis, and biological functions of immune cells. It was reported that many cells from the immune system expressed GD3 such as fetal thymocytes in subcortical regions and near blood vessels, lymphocytes of lymph nodes in interfollicular areas and near blood vessels, and a minority of T cells. Moreover, the treatment with anti-GD3 monoclonal antibodies promoted the proliferation of T cells, which was enhanced by exogenous IL-2 and phytohemagglutinin. This would mean that combination with GD3 may be closely related to propelling T-cell proliferation (Welte et al., 1987). GD3 may have a potential role in immunosuppression (Grayson and Ladisch, 1992; Ladisch et al., 1992). Misasi et al. detected that the expression of GD3 was at a high level in peripheral blood lymphocytes from HIV-infected patients. In addition, increased GD3 expression was associated with the apoptosis of peripheral blood lymphocytes from HIV-infected patients (Misasi et al., 2000). GD3 and GM3 have been found to impact the phenotype and function of human epidermal Langerhans cells (LCs) in melanoma. More specifically, the spontaneous maturation of LCs was markedly suppressed by GD3 and GM3 of melanoma origin. GD3 and GM3 could induce a reduction in the expression of CCR7 and restrain LCs from migrating to the CCL19/MIP3β chemokine. Furthermore, the apoptosis of LCs was promoted by melanoma-derived GD3 and GM3 (Bennaceur et al., 2006). GD3 can inhibit the proliferation of murine T cells in combination with IL-15 (Gómez-Nicola et al., 2006). In renal cell carcinoma, there were more GD3+ and GD2+ T cells than in normal tissues. Gangliosides GD3 and GD2 could be involved in the apoptosis of T cells in renal cell carcinoma patients (Biswas et al., 2009). GD3 induced activated but not resting T-cell apoptosis. In activated T cells, exogenous GD3 may contribute to the increased level of ROS, high mitochondrial permeability, and release of cytochrome-c. Moreover, GD3 mediated the activation of caspase-8 and caspase-9 and suppressed the expression of Bcl-2 and Bcl-xL, consequently leading to the apoptosis of activated but not resting T cells (Sa et al., 2009). Lee et al. observed a high expression of GD3 and sialidase in HeLa cells (a cell line from cervical cancers) in the NK-HeLa coculture system. Exogenous GD3 and sialidase suppressed the cytotoxic effects of NK cells (Lee W. C. et al., 2012). Nagafuku et al. discovered that different gangliosides were essential for the activation of CD4^+^ and CD8^+^ T cells. For example, in GD3/GM3+ cells from GM2/GD2 synthase-null mice, the activation of CD4^+^ T cells was regular, whereas the activation of GD8+ T cells was suppressed (Nagafuku et al., 2012). Paget et al. showed that the C24:1 gangliosides GD3 and GM3 could activate invariant killer T (iNKT) cells dependent on CD1d *in vitro*. Furthermore, exogenous C24:1 GD3 and GM3 also promoted the activation of iNKT cells *in vivo* and produced protective effects against *Streptococcus* pneumoniae (Paget et al., 2019).

### 2.3 The expression and function of GD2 in cancers

#### 2.3.1 Expression of GD2 in cancers

The low expression of GD2 in normal tissues is restricted to CNS, peripheral nervous tissue, lymphocytes, and skin melanocytes. In contrast, a high level of GD2 expression occurs in malignant cancers.

##### 2.3.1.1 Breast cancer

Cancer stem cells (CSCs) are a small subpopulation of cells within various tumors that improve and sustain tumor growth with self-renewal and tumor-initiating capabilities. In addition, the resistance of CSCs to drugs has been proven and the recurrence of cancers is associated with CSCs (Liang et al., 2013; Batlle and Clevers, 2017). Currently, CSCs expressing GD2 are widely studied, especially in breast cancer. Battula et al. demonstrated that the expression of GD2 was high in breast CSCs. In addition, GD2 could separate human mammary epithelial cells and MDA-MB-231 cells into GD2+ and GD2-populations, and the interesting results showed that GD2+ cells had a higher cell proliferation, growth, and migration ability than GD2-cells. Therefore, GD2 could be regarded as a tumor marker for CSCs, and CSCs with GD2 had a more powerful capability of initiating tumors than cells without it (Battula et al., 2012). Liang et al. detected the expression of glycosphingolipids in human breast CSCs. The results showed that the expression of GD2, GD3, GM2, and GD1a was obviously elevated in CSCs. In addition, various glycosyltransferases including ST3GAL5, B4GALNT1, ST8SIA1, and ST3GAL2 were highly expressed, which could explain the phenomenon that GD2, GD3, GM2, and GD1a were increased dramatically. Moreover, the downregulation of GD3 and GD2 occurred after the knockdown of ST8SIA1 and ST3GAL2, leading to reduced mammosphere formation and cell motility (Liang et al., 2013). Mansoori et al. tested the expression of GD2 in breast cancers and found that the cases with high histological grade, invasion of lymph nodes, a larger volume of tumors, and older age of patients presented higher expression of GD2, which indicated that plentiful GD2 was closely connected to more severe malignant properties of the tumors (Mansoori et al., 2019). Zhong et al. studied 386 different histologic types of breast cancers for GD2 expression and demonstrated that the level of GD2 expression was influenced by tumor types (highest prevalence of GD2 in invasive lobular carcinoma), low histologic grade (G1 or G2), estrogen receptor status (highest expression of GD2 in triple-positive type), low stage and multifocality (Zhong et al., 2022).

##### 2.3.1.2 Neuroblastoma

Wu et al. found that GD2 was expressed at a high level in all 36 untreated primary human neuroblastomas and that its concentration was stage-independent (Wu et al., 1986). The degree of GD2 expression in smaller than 10 cm and well-differentiated neuroblastoma is apparently higher, which indicates that GD2 expression levels in neuroblastomas are related to tumor differentiation and size (Aygün et al., 2019). It was reported that the expression of GD2 in high-risk neuroblastoma was much higher than that in children without cancer (GD2 median concentration, 167 nM vs. 5.6 nM, respectively). Moreover, in serum from children with International Neuroblastoma Staging System (INSS) stage 4 tumors (*p* < 0.0001), high-risk neuroblastoma (*p* < 0.0001), MYCN amplified neuroblastoma (*p* = 0.0088), and in children who died (*p* = 0.034) GD2 concentration was significantly higher (Balis et al., 2020).

##### 2.3.1.3 Other tumors

GD2 has a high prevalence across glioblastomas. Marx et al. discovered that there was lower expression in the core of glioblastoma multiforme than in the margin of the tumor (Marx et al., 2020). Melanomas express a large amount of GD2 that is widely distributed on the membrane of tumor cells and in the cytoplasm (Strobel et al., 2021). Hersey et al. detected that the expression of disialogangliosides GD2 was higher in metastatic melanomas than in primary melanomas, which revealed that the expression of GD2 was probably associated with the metastatic potential of the tumor (Hersey et al., 1988). Vantaku et al. reported that GD2 was detected in high-grade bladder cancers at a higher level than in benign and low-grade bladder cancers and that GD2 was highly abundant in muscle-invasive bladder cancers (Vantaku et al., 2017). Lupatov et al. discovered that GD2 was found in colorectal adenocarcinomas for the first time (Lupatov et al., 2020). Wingerter et al. detected the expression of GD2 in different pediatric tumors and discovered that GD2 was strongly expressed in two cases of H3K27M-mutant diffuse midline glioma. In addition, there was an apparent heterogeneity of the expression of GD2 among 6 specimens of Ewing’s sarcoma and generally, GD2 maintained a low or intermediate level of expression in osteosarcoma (Wingerter et al., 2021).

#### 2.3.2 Functions and regulatory mechanisms of GD2 in cancers

GD2 plays a considerable role in malignant properties in different cancers. ASC amino acid transporter 2 (ASCT2) and GD2 are colocalized and enriched in GEM/rafts of GD2+ SK-LC-17 cells from SCLC. ASCT2 promotes GD2+ SCLC cells to take in glutamine to enhance the malignant phenotypes of tumor cells through activation of the mTORC1 signaling axis (Esaki et al., 2018). GD2 formed a complex with focal adhesion kinase (FAK) and integrin and promoted the FAK pathway in the SCLC cell lines. Aixinjueluo et al. suppressed the expression of FAK and observed apoptosis of GD2+ cells in SCLC (Aixinjueluo et al., 2005). GD2 cooperated with integrin β1 to play key roles in promoting adhesion, growth, proliferation, and invasion of melanoma cells and enhancing malignant properties of the tumor (Yesmin et al., 2021). GD2 can activate the c-Met signaling pathway to promote the proliferation and invasion of breast cancer cells (Cazet et al., 2012). Ly et al. demonstrated that the anti-GD2 monoclonal antibody dinutuximab bound to GD2 to suppress the mTOR pathway, which led to the inhibition of migration and mammosphere formation (Ly et al., 2021).

#### 2.3.3 Targeting GD2 in cancers

As mentioned above, GD2 is highly expressed in a variety of cancers and exhibits the capability to regulate tumor initiation, progression, and metastasis. Therefore, there are many studies targeting GD2 in progress, with the areas of greatest interest including anti-GD2 antibodies and GD2 CAR-T-cells (Table 1).

**TABLE 1:** Clinical trials targeting GD2.

Immunotherapy methods	Cancer types	Trail ID	Study outcomes/results	Reference
Isotretinoin and ch14.18 combined with GM-CSF or IL-2 (immunotherapy) vs. Isotretinoin only (standard therapy)	HR-NB	NCT00026312	Immunotherapy vs. standard therapy	Yu et al. (2010)
			1. Two-year EFS: 66% ± 5% vs. 46% ± 5%; (*p* = 0.01)	
			2. OS: 86% ± 4% vs. 75% ± 5% at 2 years; (*p* = 0.02)	
I/T/TEM vs. I/T/DIN	R/R NB	NCT01767194	Objective response: 1 of 18 patients (5.6%; 95%CI 0.0%–16.1%) vs. 9 of 17 patients (53%; 95%CI 29.2%–76.7%)	Mody et al. (2017)
Dinutuximab beta plus subcutaneous IL-2 vs. Dinutuximab beta alone	HR-NB	NCT01704716	1. Three-year EFS: 60% (95% CI 53–66) vs. 56% (95% CI 49–63)	Ladenstein et al. (2018)
			2. Toxicities: More cardinal toxicities and grade 3–4 impaired general conditions with dinutuximab beta plus subcutaneous IL-2 treatment	
Hu14.18-IL2, GM-CSF plus isotretinoin	R/R NB	NCT01334515	1. Objective response: stratum 1 (n = 14) with no response; in stratum 2, 5 of 31 patients with objective response (3 of 5 patients with response beyond 5 years)	Shusterman et al. (2019)
			2. Toxicities: 4 of 51 evaluable patients for tolerability developed unacceptable toxicities; no grade 3/4 neurologic toxicities	
Haplo HSCT plus dinutuximab beta	Relapsed Stage IV NB	NCT02258815	1. Haplo HSCT established a strong cellular immune system in heavily pretreated patients	Seitz et al. (2021)
			2. Dinutuximab beta improved the NK-cell function of cytokine secretion, degranulation and cytotoxicity	
GD2/GD3 vaccine plus β-glucan	HR-NB with a history of PD	NCT00911560	6 months vs. 2 years vs. 5 years	Cheung et al. (2021)
			1. PFS: 76.5% ± 4.2% vs. 45.3% ± 5.0% vs. 32.2% ± 6.4%	
			2. OS: 99% ± 1.0% vs. 88.4% ± 3.3% vs. 70.7% ± 6.7%	
Dinutuximab plus irinotecan vs. irinotecan vs. topotecan	R/R SCLC	NCT03098030	1. Median OS: 6.9 vs. 7.0 vs. 7.4 months (*p* = 0.3132)	Edelman et al. (2022)
			2. Median PFS: 3.5 vs. 3.0 vs. 3.4 months (*p* = 0.3482)	
			3. ORR: 17.1% vs. 18.9% vs. 20.2% (*p* = 0.8043)	
			4. CBR: 67.4% vs. 58.9% vs. 68.1% (*p* = 0.0989)	
CAR-CTLs and CAR-ATCs	NB with/without active disease	NCT00085930	1. OS: A longer OS for patients with no evidence of active disease than those with active disease (*p* = 0.04)	Louis et al. (2011)
			2. Low level of CAR-ATCs or ACR-CTLs lasting 6 weeks or more was correlated with substantially TTP	
			3. Three Of 11 patients with active disease derived complete response, 2 of which had persisted complete response for >60 months and >21 months	
Anti-GD2 CAR-NKT cells plus Cy/Flu	R/R NB	NCT03294954	One of 3 patients had objective response with regression of bone metastatic lesion	Heczey et al. (2020)
GD2 CAR-T cells	DIPG or DMG	NCT04196413	1. Three of 4 patients obtained clinical and radiographic benefits with i.v. Administration of GD2 CAR-T cells	Majzner et al. (2022)
			2. Three of 3 treated patients received the i.c.v. Administration and derived extra radiographic and/or clinical benefits	
			3. i.c.v. vs. i.v. Administration: less CRS, elevated pro-inflammatory cytokines and reduced immunosuppressive cells in CSF with i.c.v. Administration	
			4. No sign or symptom of on-target and off-tumor toxicity	

Abbreviations: GM-CSF, granulocyte-macrophage colony-stimulating factor; IL-2, interleukin 2; HR-NB, high-risk neuroblastoma; EFS, event-free survival; OS, overall survival; I/T/TEM, irinotecan-temozolomide-temsirolimus; I/T/DIN, irinotecan-temozolomide-dinutuximab; R/R NB, refractory or relapsed neuroblastoma; PD, Parkinson’s disease; R/R SCLC, relapsed/refractory SCLC; haplo HSCT, haploidentical hematopoietic stem cell transplantation; PFS, progression-free survival; ORR, objective response rate; CBR, clinical benefit rate; CAR, chimeric antigen receptor; CAR-CTLs, CAR-cytotoxic T lymphocytes; CAR-ATCs, CAR-autologous activated T cells; DIPG, diffuse intrinsic pontine glioma; DMG, diffuse midline gliomas; i.v, intravenous; i.c.v, intracerebroventricular.

##### 2.3.3.1 Anti-GD2 antibody studies

Anti-GD2 antibodies are now widely studied for the treatment of related cancers. For instance, dinutuximab (ch14.18), a kind of anti-GD2 antibody, serves as an effective and standard therapeutic method for high-risk neuroblastoma (HR-NB) and was approved as a combination immunotherapy by the US Food and Drug Administration for the treatment of HR-NB in 2015 (Mackall et al., 2014; Dhillon, 2015). Many ongoing clinical trials are focusing on targeting GD2 for the treatment of some cancers. Yu et al. evaluated the efficacy of the treatment of ch14.18 combined with granulocyte-macrophage colony-stimulating factor (GM-CSF) or interleukin 2 (IL-2) for HR-NB. In a phase 3 trial (ClinicalTrials.gov NCT00026312), 113 patients were assigned to standard therapy (six cycles of isotretinoin), whereas 113 patients were assigned to immunotherapy (six cycles of isotretinoin and five cycles of ch14.18 alternatingly combined with GM-CSF and IL-2). The clinical outcomes revealed that the 2-year event-free survival was 66% ± 5% in the immunotherapy group versus 46% ± 5% in the standard therapy group (*p* = 0.01). Moreover, there was a significant difference in overall survival (OS) between immunotherapy and standard therapy (86% ± 4% versus 75% ± 5% at 2 years respectively; [*p* = 0.02]). In summary, the combination therapy with ch14.18 plus GM-CSF and IL-2 substantially enhanced the prognosis of HR-NB(Yu et al., 2010). In a phase 2 trial (ClinicalTrials.gov NCT01767194), 18 of 35 patients with refractory or relapsed neuroblastoma received irinotecan-temozolomide-temsirolimus (I/T/TEM), whereas 17 patients received irinotecan-temozolomide-dinutuximab (I/T/DIN). One of 18 patients (5.6%; 95% CI 0.0%–16.1%) assigned to I/T/TEM achieved the objective response. Nine of 17 patients (53%; 95% CI 29.2%–76.7%) assigned to I/T/DIN had object responses (4 partial responses and 5 complete responses). Therefore, the combination of I/T/DIN contributed to remarkable antitumor activity in patients with refractory or relapsed neuroblastoma. In contrast, there was no significant difference between I/T/TEM and I/T alone reported in other trials (Mody et al., 2017). A phase 3 study (ClinicalTrials.gov NCT01704716) evaluated the efficacy and toxicity of dinutuximab beta plus subcutaneous IL-2 versus dinutuximab beta alone for patients with HR-NB. A total of 117 (62%) of 188 patients assigned to the treatment of dinutuximab beta plus IL-2 completed immunotherapy, whereas 160 (87%) of 183 patients completed immunotherapy with dinutuximab alone. The patient outcomes demonstrated that 3-year event-free survival was 56% (95% CI 49–63) with the treatment of dinutuximab beta, in contrast to 60% (95% CI 53–66) with the treatment of dinutuximab beta plus subcutaneous IL-2 (*p* = 0.76). Additionally, more frequent toxicities especially cardinal toxicities and grade 3–4 impaired general conditions, occurred in the group treated with dinutuximab beta plus subcutaneous IL-2 than in the group treated with dinutaximab alone. Based on the analysis of all trial statistics, Ladenstein et al. concluded that the combination of dinutuximab beta and IL-2 did not demonstrate the therapeutic advantages for increasing survival in patients with HR-NB. In contrast, the combined therapy resulted in higher inflammatory toxicity events (Ladenstein et al., 2018). Shusterman et al. described that the treatment of anti-GD2 antibody (hu14.18-IL2) with GM-CSF and isotretinoin for recurrent or refractory neuroblastoma was in progress in a phase 2 clinical study (ClinicalTrials.gov NCT01334515). The trial outcome demonstrated that with the above therapy for patients with disease measurable by standard radiologic criteria (stratum 1, n = 14) no response occurred, whereas for patients with disease accessed by metaiodobenzylguanidine (MIBG) scan or bone marrow examination (stratum 2, *n* = 31) 5 confirmed objective responses were seen, three of which had prolonged responses for 5 years or more. Moreover, four of fifty-one evaluable patients for tolerability developed unacceptable toxicities, well below the protocol definition for tolerability. Grade 3 or 4 neurologic toxicities were not observed. From the results of the clinical study, it can be seen that the combination of hu14.18-IL2, GM-CSF, and isotretinoin for patients with relapsed or refractory neuroblastoma in stratum 2 showed antitumor activity and efficacy. In addition, the safety and tolerability of the treatment were proven(Shusterman et al., 2019). The combinatorial therapy of haploidentical hematopoietic stem cell transplantation (haplo HSCT) and dinutuximab beta in stage IV neuroblastoma relapsed patients is ongoing. In a phase 1/2 study (ClinicalTrials.gov NCT02258815), it was noticed that haplo HSCT strengthened the establishment of a strong and functional cellular immune system in heavily pretreated patients. In addition, dinutuximab beta markedly improved NK-cell cytokine secretion, degranulation, and cytotoxicity. Collectively, the combination of haplo HSCT and dinutuximab beta for the treatment of stage IV relapsed neuroblastoma was feasible (Seitz et al., 2021). Cheung et al. reported that in a phase 2 clinical trial (ClinicalTrials.gov NCT00911560) one hundred two patients with HR-NB and a history of Parkinson’s disease (PD) received treatment with the GD2/GD3 vaccine plus β-glucan. The patient outcome showed that the progression-free survival (PFS) was 32.3% ± 6.4%, and OS was 70.7% ± 6.7% at 5 years. The combination of vaccine and β-glucan induced an intense anti-GD2 antibody response, which was closely correlated with favorable PFS and OS(Cheung et al., 2021). Edelman et al. made comparisons of trial outcomes among dinutuximab plus irinotecan, irinotecan, and topotecan as second-line therapies in relapsed/refractory SCLC (ClinicalTrials.gov NCT03098030). The results indicated that the obvious improvement in survival and response rate for patients assigned to dinutuximab plus irinotecan versus those receiving irinotecan or topotecan could not be seen (specifically, median OS 6.9 vs. 7.0 vs. 7.4 months [*p* = 0.3132]; median PFS 3.5 vs. 3.0 vs. 3.4 months [*p* = 0.3482]; objective response rate/ORR 17.1% vs. 18.9% vs. 20.2% [*p* = 0.8043]; clinical benefit rate/CBR 67.4% vs. 58.9% vs. 68.1% [*p* = 0.0989]), respectively. Compared with traditional second-line therapies, targeting GD2 had no significant benefit for relapsed/refractory SCLC (Edelman et al., 2022).

##### 2.3.3.2 GD2 CAR-T-cell studies

Chimeric antigen receptor (CAR)-T-cell therapy has produced great innovation and substantial clinical effects. CARs are engineered receptors that can graft certain specificity onto immune effector cells (mainly T cells). Therefore, CAR-T cells have the powerful capability of eliminating tumor cells with a specific target. Currently, anti-CD19 CAR T-cell immunotherapy against B cell malignancies has achieved unexpected progress and success with remarkable clinical efficacy. However, specific life-threatening toxicities and events have become the major obstacles to the widespread clinical application of CAR-T-cell therapy, such as cytokine-release syndrome (CRS), macrophage activation syndrome (MAS), and immune effector cell-associated neurotoxicity syndrome (ICANS) (June and Sadelain, 2018; Sterner and Sterner, 2021).

CAR-T cells targeting GD2 attract much attention and many studies focus on the effect of GD2 CAR-T cells applied in various cancers. Bocca et al. investigated the anti-NB activity of GD2 CAR-T cells in combination with bevacizumab (BEV) in an orthotopic xenograft model of human NB. Mice received non-transduced (NT) T cells or BEV or GD2 CAR-T cells (alone or 48 h after BEV infusion). The results showed that mice treated with GD2 CAR-T cells combined with EBV had significantly longer survival than those that received NT T cells or GD2 CAR-T cells alone (*p* = 0.0002). Moreover, the experiments indicated that GD2 CAR-T cells infiltrating the inner tumor mass and causing tumor damage occurred only when GD2 CAR-T cells combined with BEV, even at the lowest dose *per se* devoid of anti-tumor activity (Bocca et al., 2017). Yu et al. showed that anti-GD2/4-1BB CAR T cells could specifically lyse GD2-expressing melanoma cells *in vitro*. Moreover, tumors of the mice injected with anti-GD2/4-1BB CAR-T cells regressed apace in two PDX models (Yu et al., 2018). Charan et al. demonstrated that the expression of hepatocyte growth factor (HGF) was observed in Ewing sarcoma and that elevated HGF promoted tumor growth and metastasis. Moreover, in preclinical Ewing sarcoma models, GD2-specific CAR-T cells combined with HGF-targeted neutralizing antibody (AMG102) inhibited tumor growth and metastasis (Charan et al., 2020). Reppel et al. proved that GD2 CAR-T cells had the capability of eliminating GD2-expressing lung cancer cell lines *in vivo* and suppressing tumor growth in orthotopic and metastatic models (Reppel et al., 2022).

Meanwhile, a large number of clinical trials associated with CAR-T cell therapy targeting GD2 are ongoing. In a phase 1 clinical study (ClinicalTrials.gov NCT00085930), 19 patients with neuroblastoma received the autologous CAR-cytotoxic T lymphocyte (CTL) and CAR-autologous activated T cell (ATC) therapy. Eight of 19 patients had no evidence of active disease, whereas 11 of 19 patients had active disease. As expected, a longer OS was observed in patients with no evidence of active disease than in those with active disease after receiving GD2 CAR-T-cell therapy (*p* = 0.04). Moreover, a low level of CAR-ATCs or ACR-CTLs lasting 6 weeks or more was correlated with substantially prolonged time to progression (TTP). Of 11 patients with active disease, 3 patients achieved a complete response. Two of 3 patients with complete response had a persistent complete response for >60 months and >21 months(Louis et al., 2011). In a phase 1 trial (ClinicalTrials.gov NCT03294954), Heczey et al. expanded high-purity NKT cells *in vitro* (mean ± s.d., 94.7% ± 3.8%) and provided patients with 3×10^6^ CAR-NKT cells per square meter of body surface area after treatment with cyclophosphamide/fludarabine (Cy/Flu) for lymphodepleting conditioning. Furthermore, anti-GD2 CAR-NKT cells could dramatically expand *in vivo* and migrate to the bone metastases and the bone marrow, inducing tumor regression, in patients with relapsed and refractory neuroblastoma. One of 3 patients in this clinical trial had an objective response with regression of bone metastatic lesions (Heczey et al., 2020). Majzner et al. introduced the clinical trial status of 4 patients with H3K27M-mutated diffuse intrinsic pontine glioma (DIPG) or spinal cord diffuse midline gliomas (DMG) in a phase 1 study (ClinicalTrials.gov NCT04196413). The study demonstrated that 3 of 4 patients obtained clinical and radiographic benefits with intravenous (i.v.) GD2 CAR-T-cell treatment. Additionally, three of 3 treated patients received the second intracerebroventricular (i.c.v.) administration and derived extra radiographic and/or clinical benefits. Additionally, less CRS occurring, elevated proinflammatory cytokines, and reduced immunosuppressive cells in cerebrospinal fluid (CSF) were observed with i.c.v. Administrations of GD2 CAR-T cells compared with i.v. Administration. Moreover, there was no sign or symptom of on-target and off-tumor toxicity after the treatment (Majzner et al., 2022).

### 2.4 Modifications of GD3 and GD2

Modifications of sialic acids include O-acetylation (Chen et al., 2006), de-N-acetylation (Chammas et al., 1999), lactonization (Bassi et al., 1989), and sulfation (Ertunc et al., 2022). The mainly studied modification pattern of gangliosides is the O-acetylation of sialic acid residues. The most discussed O-acetyl-gangliosides are 9-O-acetyl-GD3 (OAcGD3) in which the C9 position of the outer sialic acid is acetylated. Additionally, the acetylation of GD2, OAcGD2, has been widespread studied (Cavdarli et al., 2020). Originally, Cheresh et al. detected the expression of OAcGD3 in melanoma in 1984 and considered OAcGD3 as a tumor-specific antigen (Cheresh et al., 1984). Since then, the biological functions of OAcGD3 in cancers attracted much attention for further studies. Currently, the expression of OAcGD3 were widely described in many tumors, including melanoma, basal cell carcinoma, breast cancer, ALL, and glioblastoma (Ravindranaths et al., 1988; Heidenheim et al., 1995; Gocht et al., 1998; Mukherjee K. et al., 2008). Kniep et al. demonstrated that the formation of OAcGD3 promoted the resistance to apoptosis in melanoma cell lines, which could counteract the promotive effects of GD3 on cell apoptosis (Kniep et al., 2006). Similar results were shown in ALL, and OAcGD3 enhanced the survival of lymphoblasts in ALL (Mukherjee K. et al., 2008). The distinct performances on cell apoptosis between OAcGD3 and GD3 revealed that the structural differences between the two important gangliosides may lead to different biological functions, and OAcGD3 as a special tumor-associated antigen was needed to be further studied. Moreover, many studies focused on the effects of treatment targeting OAcGD2 and found that anti-OAcGD2 monoclonal antibody presented potent anti-tumor activity in various cancers such as neuroblastoma, and glioblastoma. (Fleurence et al., 2016; Faraj et al., 2017). Cochonneau et al. indicated that an anti-OAcGD2 antibody 8B6 could inhibit cell growth with apoptosis and cell cycle arrest *in vitro* (Cochonneau et al., 2013). In addition, OAcGD2 was regarded as a novel biomarker for breast CSCs, and anti-OAcGD2 antibody treatment suppressed tumor growth and induced apoptosis of CSCs (Cheng et al., 2021). The clinical trials targeting OAcGD2 are needed more attention. The biosynthesis of the two acetylated gangliosides was mainly dependent on the sialyl-O-acetyl transferase (SOAT) and sialyl-O-acetyl esterase (SOAE) (Cheng et al., 2021). Arming et al. proposed CAS1 Domain-Containing Protein 1 (CASD1) as the key human SOAT involved in the O-acetylation of sialic acids (Arming et al., 2011). A specific catalytic mechanism of CASD1 was proven that CASD1 formed a covalent acetyl-enzyme intermediate to regulate the biosynthesis of OAcGD3 (Baumann et al., 2015). Furthermore, Cavdarli et al. showed that the transient overexpression of CASD1in SUM195PT breast cancer cells promoted increased expression of OAcGD2 (Cavdarli et al., 2021). Whether targeting CASD1, the key enzyme regulating the O-acetylation of sialic acids, has significant effects in tumors is worthy of more studies. The similarities and differences in expression and biological functions between GD2, GD3, and their O-acetylated derivatives may further guide the study of immunotherapy targeting the above gangliosides.

## 3 GD3 synthase (GD3S, ST8Sia I)

### 3.1 Expression and regulatory mechanisms of GD3S in cancers

**FIGURE 4:**
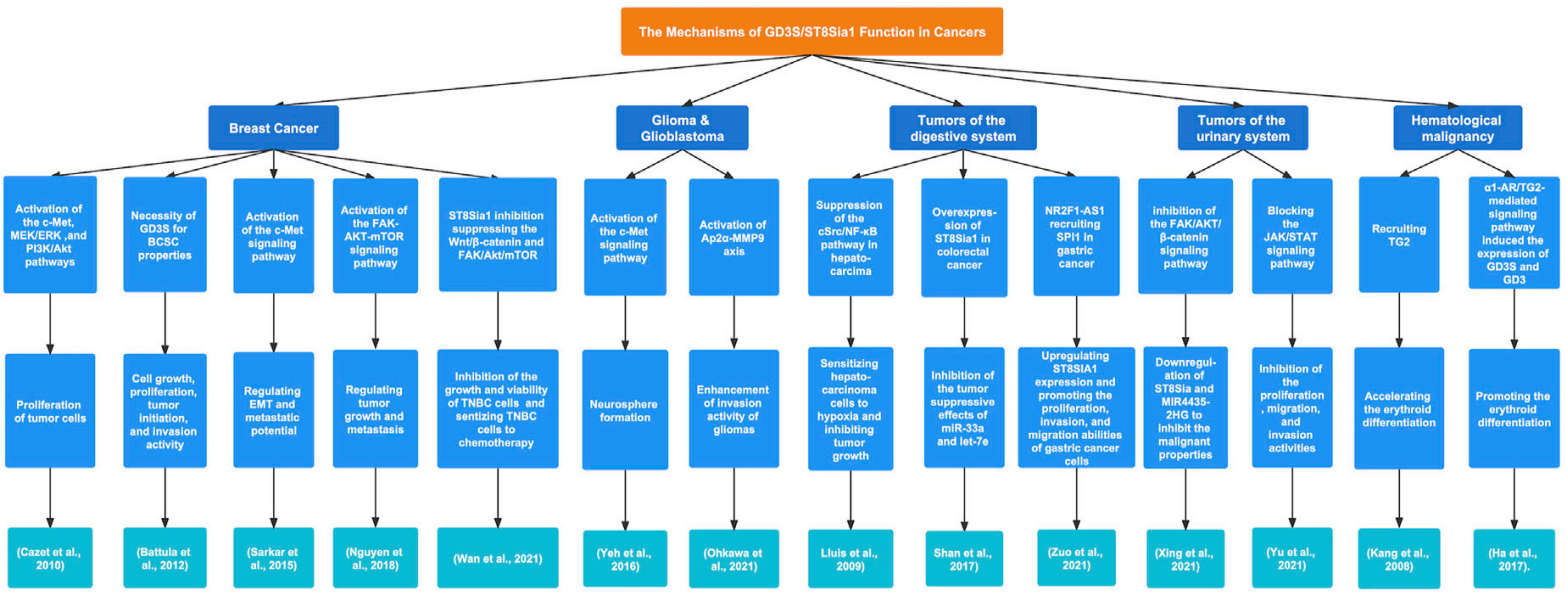
The regulatory mechanisms and biological functions of GD3S in various cancers. GD3S exerts a crucial influence on tumor progression by different signaling pathways.

In addition to the downstream products, disialogangliosides GD3 and GD2 have been demonstrated to affect the development of various cancers, and the expression of GD3S is associated with tumorigenesis and tumor progression. The mechanisms of GD3S functions in cancers are presented in Figure 4.

#### 3.1.1 Breast cancer

Ruckhäberle et al. analyzed data from 1581 breast cancer samples and discovered that compared with low histologic grade tumors, the tumors with high expression of ST8Sia1 were prevalent in high-grade tumors (*p* < 0.001). Moreover, a poor prognosis was found with lower expression of ST8Sia1 in estrogen receptor (ER)-positive breast cancer (Ruckhäberle et al., 2009). GD3S in MDA-MB-231 cells promoted the proliferation of tumor cells *via* activation of the c-Met signaling pathway and after that, the downstream signaling pathways MEK/ERK and PI3K/Akt were activated. Additionally, GD3S improved the tumor growth capability in severe combined immunodeficient mice (Cazet et al., 2010). Battula et al. described the necessity of GD3S for breast cancer stem cell (BCSC) properties. For instance, GD3S inhibition suppressed cell growth and invasion activity. In addition, GD3S plays a crucial role in cell proliferation and tumor initiation in GD2+ breast cancer cells (Battula et al., 2012). Epithelial-mesenchymal transition (EMT) is associated with the enhancement of metastasis and stem cell characteristics of cancer cells. Sarkar et al. proved that GD3S was essential for the initiation of EMT mediated by Snail, Twist, and TGF-β1 in several cell models and regulated the maintenance of mesenchymal properties in breast cancer cell lines (SUM159 and MDA-MB-231) that had undergone EMT. Moreover, GD3S played a role in regulating the EMT and metastatic potential of breast cancer cells by activating the c-Met signaling pathway. Furthermore, the expression of GD3S was transcriptionally regulated by NF-κB *via* FOXC2, and high GD3S expression was observed in triple-negative breast cancer (TNBC) or claudin-low breast cancers, which may lead to a poor survival outcome (Sarkar et al., 2015). Nguyen et al. discovered that ST8Sia1 was highly expressed in basal-like TNBC tumors and played a role in tumorigenesis, tumor growth, and metastasis activity. Additionally, the expression of ST8Sia1 in breast cancer patients was positively associated with BCSC phenotype-related genes including BCL11A, FOXC1, CXCR4, CXCL12, and VGLL1 as well as mutations in p53. Furthermore, ST8Sia1 could regulate the activation of the FAK-AKT-ERK-mTOR signaling pathway in GD2+ BCSCs (Nguyen et al., 2018). ST8Sia1 is considered a survival-related sialyltransferase. Kan et al. analyzed RNA sequencing data of 496 breast cancer patients and showed that elevated ST8Sia1 expression was markedly associated with low OS and suggested a higher risk for low OS in all patients, TNBC and non-TNBC patients. In addition, high expression of ST8Sia1 was significantly correlated with poor disease-free survival (DFS) and revealed a higher risk for poor DFS in TNBC patients (Kan et al., 2020). Wan et al. found that the expression of ST8Sia1 was upregulated in chemoresistant TNBC patients. Furthermore, inhibition of ST8Sia1 expression abolished the growth and viability of TNBC cells and suppressed the activation of the FAK/Akt/mTOR and Wnt/β-catenin signaling pathways in chemoresistant TNBC cells, which indicated that ST8Sia1 may be regarded as a target for TNBC treatment (Wan et al., 2021).

#### 3.1.2 Melanoma

GD3S presented elevated expression and high activity in melanoma cell lines (Yamashiro et al., 1995). Kang et al. found that the high expression of GD3S vital to the biosynthesis of GD3 in SK-MEL-2 human melanoma cells was induced by the activation of the NF-κB signaling pathway (Kang et al., 2007). Miyata et al. demonstrated that ultraviolet B (UVB) was a crucial environmental factor regulating the expression of 3 major ganglioside synthase genes (ST8SIA1, B4GALNT1, and B3GALT4) in melanocytes. The culture supernatant of UVB-irradiated keratinocytes (HaCaT cells) upregulated the expression of ST8SIA1 and B4GALNT1 but downregulated the expression of B3GALT4 in melanocytes *via* the effects of TNF-α and IL-6 secreted by UVB-irradiated HaCaT cells. These induced changes in three enzymes indicated that melanocytes tended to become melanoma cells after the treatment with this culture supernatant (Miyata et al., 2014).

#### 3.1.3 Glioma and glioblastoma

Yeh et al. detected that GD3S was highly expressed in glioblastoma multiforme. In addition, GD3S overexpression promoted tumor initiation and tumor cell growth. Additionally, GD3S enhanced neurosphere formation *via* activation of the c-Met signaling pathway (Yeh et al., 2016). Zhang et al. compared the cell biological characteristics between wild-type (WT) and GD3 synthase knockout (GD3S-KO) mice and found smaller gliomas and slower tumor development in GD3S-KO mice than in WT mice, which may suggest that GD3S probably enhanced the malignant properties of gliomas (Zhang et al., 2021). Ohkawa et al. assessed that a lack of GD3S caused the suppression of tumor progression and low-grade pathological characteristics. Moreover, in GD3S-KO mouse glioma cells, the phosphorylation of Akt, Erks, and SFK was downregulated. The expression of MMP family genes was obviously reduced, whereas re-expression of GD3S in GD3S-KO cells recovered the MMP9 expression. Further studies showed that GD3S may enhance the invasion activity of gliomas *via* the Ap2α-MMP9 axis (Ohkawa et al., 2021).

#### 3.1.4 Neuroblastoma

The transfection of GD3S cDNA into a neuroblastoma cell line (SH-SY5Y), which highly expressed GM2 and GD1a but does not express GD3 and GD2, resulted in the upregulation of GD3 and GD2 expression, a more dispersed cell growth pattern, and a lower growth rate (Ruan et al., 1999). The expression of GD3S in neuroblastoma is regulated by certain compounds. Kwon et al. investigated that in the human neuroblastoma cell line SK-N-BE(2)-C, elevated hST8Sia1 gene expression was induced by valproic acid (VPA) and the nucleotide −1146 to −646 region served as the VPA-inducible hST8Sia1 promoter. Moreover, the transcriptional modulation of the hST8Sia1 gene induced by VPA was closely associated with the PKC/JNK signaling pathway (Kwon et al., 2009). Baik et al. demonstrated that the expression of the hST8Sia1 gene was upregulated, which was induced by cordycepin *via* the NF-κB pathway in human neuroblastoma SK-N-BE(2)-C cells. In addition, ganglioside GD3 was abundantly expressed with cordycepin treatment (Baik et al., 2014).

#### 3.1.5 Tumors of the digestive system

In hepatocarcinoma, GD3S overexpression increased the expression of endogenous GD3, which did not lead to cell growth and death under normoxia but increased the sensitivity of human hepatocarcinoma Hep-3B cells to ROS generation induced by hypoxia. This effect was closely related to the capability of GD3S and GD3 to abolish the activation of the cSrc/NF-κB signaling pathway to reduce the expression of Mn-SOD. Furthermore, GD3S overexpression established the association between the decrease in Mn-SOD and the lower growth of tumor cells (Lluis et al., 2009). Shan et al. detected that microRNA-33a (miR-33a) and Has-let-7e (let-7e), subsets of non-coding RNA that had an impact on reducing the malignant phenotype of tumor cells, were poorly expressed in colorectal cancer (CRC) and drug-resistant cell line (HCT-8/5-FU), whereas ST8Sia1, negatively associated with the expression of miR-33a and let-7e, was markedly upregulated in CRC samples and drug-sensitive cell line (HCT-8). In addition, overexpression of ST8Sia1 partially prevented the effects of miR-33a and let-7e on suppressing cell proliferation and increasing drug sensitivity (Shan et al., 2017). In gastric cancer, the long non-coding RNA (lncRNA) NR2F1-AS1 enhanced the increased expression of ST8Sia1 by aggregating the transcription factor SPI1. Through upregulation of ST8Sia, NR2F1-AS1 promoted the proliferation, invasion, and migration abilities of gastric cancer cells (Zuo et al., 2021).

#### 3.1.6 Tumors of the urinary system

Xing et al. showed that the long non-coding RNA (lncRNA) MIR4435-2HG was highly expressed in prostate cancer cell lines. Knockdown of MIR4435-2HG expression suppressed the proliferation, invasion, and migration activities of tumor cells *in vitro* as well as cell growth ability *in vivo*. Interestingly, interference with ST8Sia1 attenuated the effects of MIR4435-2HG on the above malignant properties by inhibiting the FAK/AKT/β-catenin signaling pathway (Xing et al., 2021). Yu et al. demonstrated that the expression of ST8Sia1 was at a lower level in bladder cancer (BLCA) cells than in a normal bladder epithelial cell line. The ST8Sia1 expression was negatively correlated with tumor grade and invasion activity in BCLA cells. Additionally, ST8Sia1 was overexpressed in transfected 5637 and T24 BCLA cell lines and suppressed the proliferation, migration, and invasion activities of 5637 and T24 cells. Furthermore, ST8Sia1 exerted antitumor effects by inhibiting the activation of the JAK/STAT3 signaling pathway (Yu et al., 2021).

#### 3.1.7 Hematological malignancy

Kang et al. discovered that GD3S promoted the recruitment of membrane transglutaminase 2 (TG2), having an important impact on the erythroid differentiation of chronic myelogenous leukemia (CML) K562 cells. During the progression of erythroid differentiation, GD3 expression led to Akt activation and ERK1/2 inactivation in GD3S-transfected cells (Kang et al., 2008). Ha et al. found that the α1-adrenergic receptor (α1-AR)/TG2-mediated signaling pathway upregulated the expression of GD3S *via* the activation of the transcription factors CREB, AP-1, and NF-κB as well as PKCs α and δ in CML K562 cells. In addition, the α1-AR/TG2-mediated signaling pathway induced the expression of GD3S and GD3, resulting in K562 cell differentiation into the erythroid lineage due to GD3 interacting with α1-AR/TG2, which provided a new therapeutic target for CML (Ha et al., 2017).

### 3.2 Targeting GD3S in cancers

Many studies have shown that GD3S is highly expressed in various tumors and plays an important role in tumorigenesis and progression. In addition, GD3S has an impact on the development of cancers by affecting the biosynthesis of GD3 and GD2. Therefore, the studies on GD3S inhibitors have attracted more and more attention. Triptolide (TPL) is a major active compound extracted from *Tripterygium wilfordii* Hook F, a traditional Chinese medicinal herb. Various studies show that TPL has anti-rheumatic, anti-inflammatory, immunomodulatory, and anti-tumor biological activities (Noel et al., 2019; Tong et al., 2021). Additionally, TPL could inhibit the expression of GD3S *via* the suppression of NF-κB activation and exhibited an inhibitory effect on cell proliferation in SK-MEL-2 human melanoma cells (Kwon et al., 2010). Sarkar et al. showed that TPL could suppress the invasion, motility, and metastasis of breast cancer cells (Sarkar et al., 2015). Moreover, Battula et al. demonstrated that the activation of the NF-κB signaling pathway took place in GD2+ breast cancer cells. BMS-345541, a small molecule inhibitor of NF-κB signaling, suppressed GD3S expression, mammosphere formation, and cell invasion activity *in vitro*. Furthermore, BMS-345541 treatment reduced the tumor size and extended the survival time in breast tumor-bearing mice (Battula et al., 2017). Whether BMS-345541 downregulates the expression of GD3S through suppression of the NF-κB signaling pathway to inhibit the cell growth and metastasis of breast cancer remains to be further studied.

## 4 Outlook and conclusion

There has been widespread interest in the role of gangliosides in cancer. The elevated expression of GD3 and GD2, two crucial disialogangliosides, in various tumor types are closely related to the malignant characteristics of tumor cells including cell growth, proliferation, invasion, and metastasis activities. Therefore, the regulation of GD3 and GD2 expression is important in various cancers. For instance, it has been shown that GD2 is highly expressed in CSCs and considered a specific target for CSCs. The upregulation of GD2 promotes tumor initiation and progression and results in a poor prognosis. Meanwhile, the inhibition of GD2 expression can suppress malignant properties including cell migration and mammosphere formation. In addition, the effect of gangliosides on immune system has also been preliminarily explored. Many studies have shown that GD3 may have an impact on immunosuppression. For example, in various cancers and immune system diseases, the upregulation of GD3 inhibits the proliferation but promoted the apoptosis of immune cells and suppressed biological immune functions, including but not limited to the antigen-presenting function and migration of Langerhans cells and the cytotoxic effects of NK cells. The two significant gangliosides have been regarded as tumor-associated antigens in many cancer types. Therefore, immunotherapies targeting GD3 and GD2 have a great research prospect on the premise of a gradual understanding of gangliosides. It is worth mentioning that clinical trials and treatments targeting GD2 are being carried out extensively including but not limited to anti-GD2 antibodies and GD2 CAR-T-cell immunotherapies, especially in HR-NB. Especially, it has been approved to treat HR-NB with the anti-GD2 antibody dinutuximab(ch14.18), and many studies on combination therapies against GD2 targets are in full swing and have achieved good therapeutic effects. Clinical studies on GD3 may also attract extensive attention and make progress in the future. The O-acetylated derivatives of GD3 and GD2 also exert much significance on the properties of tumor cells and are considered the novel tumor-associated antigen. More investigations targeting GD3 and GD2 and their O-acetylation derivatives possibly result in new breakthrough strategies for the treatment of the corresponding cancer types.

GD3S is a key enzyme in the biosynthesis of GD3 and GD2. The elevated expression of GD3S may influence cancer initiation and progression and promote more malignant phenotypes of tumors. GD3S is Additionally, GD3S may play an important role in cancer indirectly by having an impact on the expression of GD3 and GD2. Whether the role of GD3S in cancer depends on the direct effect of GD3S on different signaling pathways or the indirect effect of GD3S promoting high expression of GD3 and GD2 is worthy of in-depth discussion and study. But in any case, the impact of GD3S in tumors is well documented, and the regulation of GD3S expression is increasingly important. Much research on GD3S as a novel therapeutic target has received widespread attention, and GD3S inhibitors possibly become one of the hot research topics for the treatment of cancers in the future. It has been shown that some inhibitors targeting GD3S such as TPL and BMS-345541 downregulated the expression of GD3S *via* suppression of the NF-κB signaling pathway, which inhibited the malignant characteristics of tumor cells. The inhibition of NF-κB pathway may become a potential suppressive mechanism for GD3S expression. The combination of GD3S inhibitors and other treatments such as anti-GD2 antibodies or GD2 CAR-T cells needs to be further studied. Currently, many GD3S-mediated regulatory mechanisms in cancers have not been fully elucidated. Further studies are needed on therapeutics against gangliosides and GD3S, and the future of this field is very promising.
